# Hepatic microcirculatory disturbance in liver diseases: intervention with traditional Chinese medicine

**DOI:** 10.3389/fphar.2024.1399598

**Published:** 2024-07-23

**Authors:** Pei Liu, Wan-Li Liang, Rui-Ting Huang, Xin-Xing Chen, De-Hua Zou, Hiroshi Kurihara, Yi-Fang Li, You-Hua Xu, Shu-Hua Ouyang, Rong-Rong He

**Affiliations:** ^1^ State Key Laboratory of Quality Research in Chinese Medicine, Macau University of Science and Technology, Macau SAR, China; ^2^ Guangdong Engineering Research Center of Traditional Chinese Medicine & Disease Susceptibility, Guangdong-Hong Kong-Macao Universities Joint Laboratory for the Internationalization of Traditional Chinese Medicine, Guangzhou Key Laboratory of Traditional Chinese Medicine & Disease Susceptibility, International Cooperative Laboratory of Traditional Chinese Medicine Modernization and Innovative Drug Development of Chinese Ministry of Education (MOE), Guangdong Province Key Laboratory of Pharmacodynamic Constituents of TCM and New Drugs Research, State Key Laboratory of Bioactive Molecules and Druggability Assessment, Jinan University, Guangzhou, China

**Keywords:** hepatic microcirculatory disturbance, liver disease, pathogenesis, traditional Chinese medicine, active metabolite

## Abstract

The liver, a complex parenchymal organ, possesses a distinctive microcirculatory system crucial for its physiological functions. An intricate interplay exists between hepatic microcirculatory disturbance and the manifestation of pathological features in diverse liver diseases. This review updates the main characteristics of hepatic microcirculatory disturbance, including hepatic sinusoidal capillarization, narrowing of sinusoidal space, portal hypertension, and pathological angiogenesis, as well as their formation mechanisms. It also summarized the detection methods for hepatic microcirculation. Simultaneously, we have also reviewed the characteristics of microcirculatory disturbance in diverse liver diseases such as acute liver failure, hepatic ischemia-reperfusion injury, viral hepatitis, non-alcoholic fatty liver disease, hepatic fibrosis, hepatic cirrhosis, and hepatocellular carcinoma. Finally, this review also summarizes the advancement in hepatic microcirculation attributed to traditional Chinese medicine (TCM) and its active metabolites, providing novel insights into the application of TCM in treating liver diseases.

## 1 Introduction

The liver is a crucial metabolic organ within the human body, serving various physiological functions, including macronutrient metabolism, blood volume regulation, blood sugar regulation, immune system support, endocrine control of growth signaling pathways, lipid and cholesterol homeostasis, and the breakdown of xenobiotic metabolites (Trefts et al., 2017; Zhang et al., 2019). The liver’s microcirculatory system comprises structures such as the hepatic artery, portal vein, hepatic sinusoids, and central vein. It features a unique dual blood supply system, with blood sourced from terminal branches of the portal vein and hepatic artery flow into the hepatic sinusoids, accompanied by the hepatic bile duct and lymphatic vessels, ultimately flowing into the central vein (Burchill et al., 2019; Torres Rojas and Lorente, 2023). Hepatic microcirculation, the crucial role in the overall physiology and function of the whole organism, supplies oxygen and nutrients to the substantial tissues and clears toxicants and foreign bodies from the bloodstream. However, various factors such as emotional stress (Li et al., 2013), medication (Wang et al., 2020), alcohol (Han et al., 2023) and virus (Orabueze et al., 2024) can disrupt the complex microcirculation in the liver, leading to liver ischemia, hypoxia, and metabolic disturbance, which in turn leads to diseases such as acute liver failure (ALF) (Gurakar et al., 2024), non-alcoholic fatty liver disease (NAFLD) (Pan et al., 2021), alcoholic liver disease (ALD) (Han et al., 2023), viral hepatitis (Kao et al., 2021), hepatic cirrhosis (Davies et al., 2017), and hepatocellular carcinoma (HCC) (Rumgay et al., 2022). Therefore, improving hepatic microcirculation has become a promising way to prevent and treat liver diseases.

At present, western medical approaches in treating hepatic microcirculatory disturbance include drug therapy and surgical treatment, yet all these methods possess certain limitations and side effects. In terms of drug therapy, vasoactive modulators or anticoagulants are the first choice to treat hepatic microcirculation, such as β Receptor blockers, rivaroxaban, and aspirin, which can cause symptoms like dizziness, hypotension, and gastrointestinal bleeding (Guixé-Muntet et al., 2020; Zhang et al., 2022). Surgical treatment, such as interventional therapy based on catheters, like transjugular intrahepatic portosystemic shunt (Jin and Zhang, 2024), can improve liver blood flow and alleviate portal hypertension but also come with risks like bleeding, infection, and embolism. Surgical removal (Pan et al., 2024) or transplantation of the liver (Lieber et al., 2024) could eliminate diseased tissue or restore liver function. Nevertheless, these surgical procedures carry significant risks, including trauma, bleeding, infection, and rejection. Hence, implementing secure and potent strategies to ameliorate hepatic microcirculation is critical for preventing and treating liver diseases. Traditional Chinese medicine (TCM) possesses multi-metabolites, multi-targets, and multi-pathways characteristics that comprehensive regulation and personalized treatment of patients, with significant advantages in regulating hepatic microcirculatory disturbance (Han et al., 2017). In this review, we aim to summarize the pathological characteristics and detection methods of hepatic microcirculatory disturbance, the characteristics of hepatic microcirculatory disturbance in diverse liver diseases as well as the potential therapeutic effects of TCM on treating diverse liver diseases by modulating hepatic microcirculatory disturbance, providing reference and inspiration for clinical and scientific research.

## 2 Characteristics of hepatic microcirculatory disturbance

**FIGURE 1:**
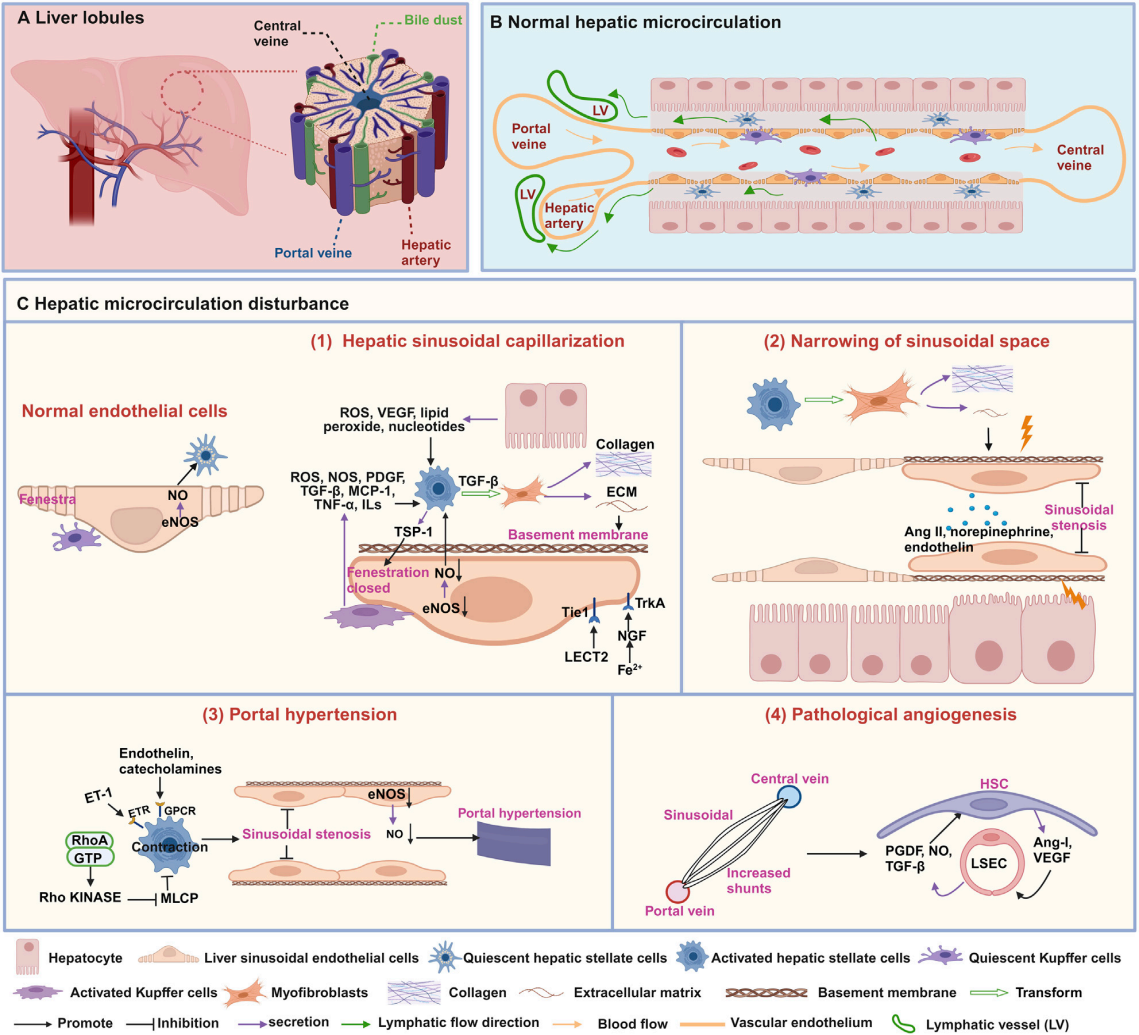
Structures and characteristics of normal hepatic microcirculation and hepatic microcirculatory disturbance. **(A)** Schematic diagram of the liver composed of hepatic lobules. **(B)** Normal hepatic sinusoidal microcirculation. **(C)** characteristics of hepatic microcirculatory disturbance. (1) Hepatic sinusoidal capillarization, (2) narrowing of sinusoidal space, (3) portal hypertension, and (4) pathological angiogenesis. This figure is created with biorender.com.

The hepatic microcirculation is a highly complex and coordinated system that involves the synergistic effect of various cell types, such as hepatic stellate cells (HSCs), liver sinusoidal endothelial cells (LSECs), and Kupffer cells (KCs) (Gracia-Sancho et al., 2019). Its main goal is to maintain the homeostasis of liver metabolism and immune function (Figures 1A, B). However, when hepatic microcirculation experiences disruption, it can develop characteristics such as hepatic sinusoidal capillarization (Zhang et al., 2022), narrowing of sinusoidal space (Mitten et al., 2023), portal hypertension (Iwakiri and Trebicka, 2021), and pathological angiogenesis (Li et al., 2023). Therefore, a deeper understanding of these characteristics can help reveal the complexity of hepatic microcirculation and provide crucial insights into the pathogenesis of liver diseases.

### 2.1 Hepatic sinusoidal capillarization

The hepatic sinusoid is a particular type of capillary located between adjacent liver plates, which is the histological basis for normal material exchange between blood and liver cells. Its inner wall has a layer of LSECs, the liver’s highest proportion of non-parenchymal cells (Gracia-Sancho et al., 2021). The normal morphology and function of the hepatic sinusoid play a crucial role in maintaining liver physiological function. Prolonged damage may result in hepatic sinusoidal capillarization, marked by the loss of fenestrae in LSECs and the formation of an endothelial basement membrane (Zheng et al., 2020) (Figure 1C-1). The phenomenon of hepatic sinusoidal capillarization occurs in a variety of liver diseases, including hepatic fibrosis (Zhang et al., 2022), cirrhosis, and HCC (Fu et al., 2023). Therefore, an in-depth understanding of hepatic sinusoidal capillarization is crucial for understanding liver disease pathogenesis.

Fenestrae are distinctive structures in LSECs, with diameters ranging from 50 to 100 nm, creating relatively wide intercellular gaps that allow passive transport of solutes, large molecules, and particles between the hepatic sinusoidal lumen and hepatocytes (Shetty et al., 2018). Various factors, including drugs, hormones, diseases, and aging, can impact the diameter, porosity, and frequency of fenestrations in LSECs (Szafranska et al., 2021). Studies have shown that activated KCs can secrete various signaling molecules such as reactive oxygen species (ROS), nitric oxide synthase (NOS), platelet-derived growth factor (PDGF), transforming growth factor (TGF)-β, monocyte chemoattractant protein-1 (MCP-1), tumor necrosis factor-α (TNF-α), and interleukins (ILs) to induce the activation of HSCs (Yang et al., 2009; Yoshizawa et al., 2022; Blas-García and Apostolova, 2023). Furthermore, the release of ROS, vascular endothelial growth factor (VEGF), lipid peroxide, TGF-β, PDGF and ILs from hepatocytes has also reported to induce the activation of HSCs. Subsequently, HSCs are converted into myofibroblasts, producing collagen and extracellular matrix (ECM), leading to a decrease or disappearance of fenestrae in LSECs (Blas-García and Apostolova, 2023). Thrombospondin-1 (TSP-1) secreted by activated HSCs play a pivotal role in regulating the contractile force and tension of LSECs through cytoskeletal signaling pathways, ultimately resulting in the closure and disappearance of fenestrae (Venkatraman and Tucker-Kellogg, 2013). Moreover, CD47-mediated downregulation of the endothelial nitric oxide synthase-nitric oxide (eNOS-NO) signaling pathway inhibits nitric oxide (NO) levels and induces contraction of LSECs, thereby leading to disappearance of fenestrae (Koch et al., 2021; Bian et al., 2022). Leukocyte cell-derived chemotaxin 2 (LECT2) bind to the tyrosine kinase receptor (Tie1) on the surface of LSECs, affecting the quantity and size of fenestrae (Du and Wang, 2022). During the early stages of hepatic sinusoidal capillarization, activated phagocytic cells migrate into the hepatic sinusoids, causing narrowing or obstruction of the sinusoids and reducing the number of fenestrae in LSECs, as well as decreasing fenestrae diameter (Cai et al., 2021). Furthermore, hepatocytes in iron-overloaded mice exhibit high expression of nerve growth factor (NGF), which actively induces the closure of fenestrae in LSECs by interacting with the NGF receptor Tropomyosin receptor kinase A (TrkA) (Addo et al., 2015).

A basement membrane between endothelial cells and hepatocytes is absent in normal liver tissue, providing high permeability to LSECs. The formation of basement membrane may result from HSCs activation and ECM deposition during the liver injury (Ortiz et al., 2021). Laminin and collage Type IV, the main components of the basement membrane, are deposited in the space of Disse. Nidogen-1 is mainly deposited in the parenchymal area around the portal ducts, and perlecan is mainly distributed in the basement membrane of the portal bile ducts and vascular structures and the hepatic sinusoidal wall. Other minor basement membrane molecules, such as Col XVIII, can be found in the space of Disse (Mak and Mei, 2017).

Research has demonstrated that numerous medications actively regulate hepatic sinusoidal capillarization to exert hepatoprotective effects. For example, Hunt et al. (2019) found that NO donors, including sildenafil, amlodipine, and nitroglycerin, can increase the porosity of LSECs. Through the eNOS and AMP-activated protein kinase pathways, metformin sulfate may improve insulin resistance, increase the porosity of LSECs fenestrae in both young and elderly animals, and slow down the aging process (Hunt et al., 2020).

### 2.2 Narrowing of sinusoidal space

**FIGURE 2:**
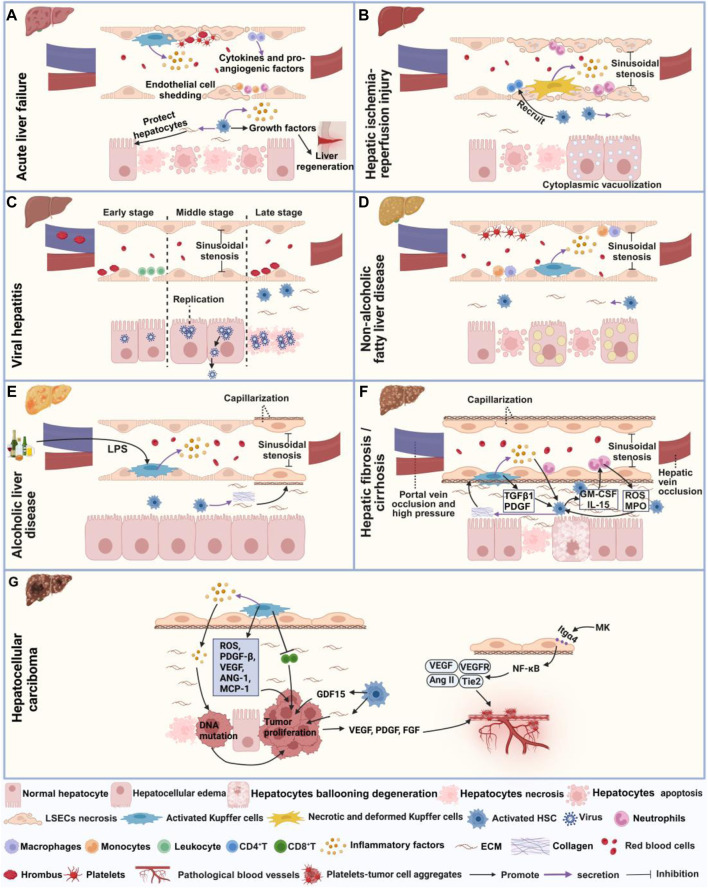
Microcirculatory disturbance in liver diseases. **(A)** Shows microcirculatory disturbance in acute liver failure; **(B)** Shows microcirculatory disturbance in hepatic ischemia-reperfusion injury; **(C)** Shows microcirculatory disturbance in viral hepatitis, **(D)** Shows microcirculatory disturbance in non-alcoholic fatty liver disease; **(E)** Shows microcirculatory disturbance in alcoholic liver injury; **(F)** Shows microcirculatory disturbance in hepatic fibrosis and cirrhosis, and **(G)** Represents microcirculatory disturbance in hepatocellular carcinoma. This figure is created with biorender.

Under normal circumstances, the diameter of hepatic sinusoids remains relatively stable, ensuring sufficient blood flow through the liver to maintain normal physiological function. However, when the sinusoidal space narrows, this passage becomes restricted, impeding blood flow (Mitten et al., 2023). Narrowing of sinusoidal space is a common pathological feature observed in viral hepatitis (Maini and Peppa, 2013), NAFLD (Mitten et al., 2023), and cirrhosis (Shenoda and Boselli, 2019). Research suggests that fibrosis regulation and contractile factors play a critical role in narrowing of sinusoidal space (Figure 1C-2). Activated HSCs release the ECM, leading to fibrosis and scar formation, and they mechanically compress the hepatic sinusoids, narrowing of sinusoidal space (Shenoda and Boselli, 2019). Vasoconstrictors, such as norepinephrine, angiotensin II (Ang II), and endothelin, can also cause constriction of the vessels surrounding the hepatic sinusoids and the small branches of the hepatic veins, thereby reducing the diameter of the hepatic sinusoids (Shenoda and Boselli, 2019; Rajapaksha et al., 2021).

Research on drug formulations for widening the hepatic sinusoidal spaces still needs to be completed. The primary solution is to address the root causes of hepatic sinusoidal space constriction, such as vascular obstruction. For instance, sildenafil can reduce platelet aggregation markers CD41 and P-selectin, mitigate LSECs dysfunction and endothelial barrier damage associated with sinusoidal obstruction syndrome, thereby improving blood hypercoagulability and contributing to alleviating hepatic sinusoidal stenosis (Mansour. et al., 2021).

### 2.3 Portal hypertension

**FIGURE 3:**
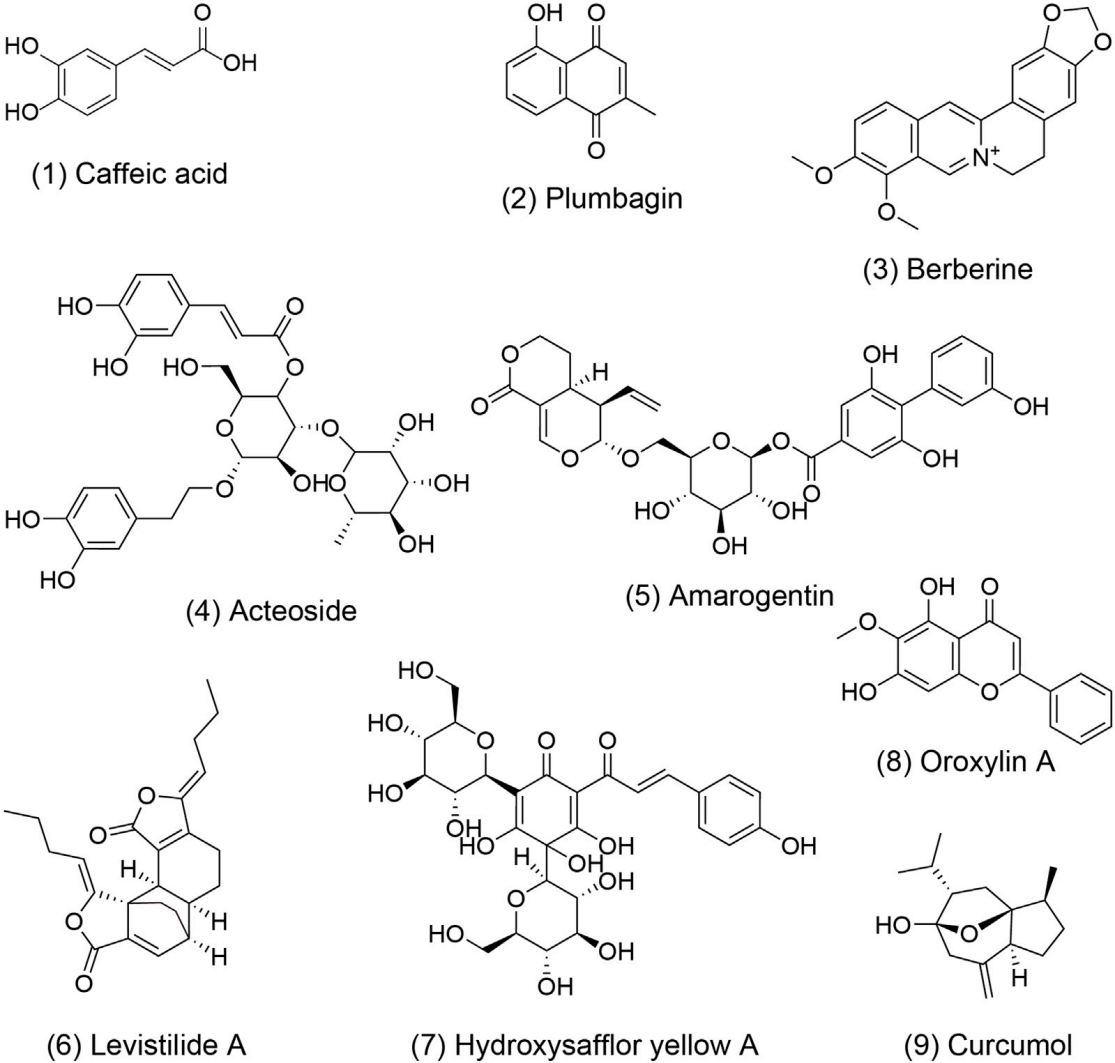
The molecular structure of metabolites with activity to improve hepatic microcirculatory disturbance. The numbers within parentheses correspond to the numbers in the main text and table. These chemical structures were plotted using ChemBioDraw Ultra 14.0.

Portal hypertension is an essential manifestation of microcirculation disturbance in diseases such as NAFLD (Baffy, 2018), hepatic fibrosis (Torres Rojas and Lorente, 2023), cirrhosis (Iwakiri and Trebicka, 2021), and is also one of the main complications of chronic liver disease. The primary cause of portal hypertension is a pathological increase in intrahepatic vascular resistance (Gracia-Sancho et al., 2019). The activation of HSCs and the function of LSECs directly contribute to the formation of portal vein hypertension (Figure 1C-3). Research has found that impaired function of LSECs results in decreased of NO synthesis, disappearance of fenestrae, increase synthesis of microvilli, adhesion molecule and basement membrane, resulting in a decrease in hepatic sinusoidal permeability, changes in hemodynamics and vascular tension, ultimately leading to the development of portal hypertension (Schierwagen et al., 2020; Iwakiri and Trebicka, 2021). Additionally, portal hypertension is also related to HSCs contraction, which is mainly due to factors such as endothelin and catecholamines acting on G protein-coupled receptors (GPCRs) or the RhoA/Rho kinase pathway, inhibiting myosin light chain phosphatase (MLCP) to further constrict HSCs, thus leading to hepatic sinusoidal stenosis and inducing portal hypertension (Iwakiri and Trebicka, 2021).

Nowadays, research has demonstrated that some medications, including tofogliflozin, possess the effects to suppressed Ac-HSC-stimulated capillarization and vasoconstriction in LSECs by enhancing the antioxidant capacity, as well as to inhibit the capillaries LSEC-stimulated contractive, profibrogenic and proliferative activities of Ac-HSCs, thus prevention portal hypertension (Asada et al., 2024).

### 2.4 Pathological angiogenesis

Angiogenesis is the dynamic process of generating new blood vessels from existing vessels, and this microcirculatory disturbance mainly occurs in HCC (Fu et al., 2023). There are two primary modalities: budding and invagination. Either way, it requires the formation of a lumen in the nascent vessel and stabilization of the nascent vessel by structures such as the basement membrane, smooth muscle cells, and peripheral nerves (Thabut and Shah, 2010). Evidence suggests that LSECs and HSCs are crucial to hepatic angiogenesis (Figure 1C-4). HSCs can directly wrap around newly formed blood vessels, providing stability and durability to prevent the collapse or degradation of blood vessels composed by LSECs. Conversely, HSCs can release pro-angiogenic substances like VEGF and angiopoietin-1, which activate LSECs to create a favorable sinusoidal environment for angiogenesis. Moreover, LSECs can also release NO, TGF-β, and PDGF to encourage HSCs migration toward neovascularization (Thabut and Shah, 2010).

Several drugs have demonstrated the ability to inhibit abnormal neovascularization. Sorafenib, for instance, reduces angiogenesis by inhibiting hypoxia-inducible factor-1α (HIF-1α) and VEGF protein expression (Liu et al., 2012). By interfering with the vascular supply, lidocaine can prevent neovascularization (Suzuki et al., 2020).

## 3 Detection methods for hepatic microcirculation

The microstructure and blood flow influence the liver’s overall function. Any abnormalities in the liver’s structure or blood flow can directly impact its blood supply and oxygen delivery, impairing its function. Consequently, employing specific methods to examine the microstructure and blood flow of the liver holds excellent significance in gaining insights into the liver’s pathophysiological processes.

### 3.1 Hematoxylin and eosin (H&E) staining

Pathological examination is a crucial detection technique that permits the observation of alterations in tissue or cell morphology, structure, and function. It facilitates the evaluation of the extent and mechanisms of hepatic microcirculatory changes by examining the steady state of the liver’s microstructure. H&E staining (Cardiff et al., 2014) is one of the crucial methods for diagnosing tissue diseases, as well as simple staining techniques by using two dyes, hematoxylin and eosin, to label the nuclei and cytoplasm of liver tissue. Using this technique, researchers could observe the structure of liver lobules, including hepatocytes, central veins, portal veins, and hepatic artery branches (Boyd et al., 2020). However, H&E staining only focuses on cellular morphological features without providing information regarding intracellular molecular expression and function. Furthermore, it cannot differentiate between components such as proteins, nucleic acids, or polysaccharides within tissues. Additionally, it is unable to depict cellular organelle structures and to be proceed with the *in vivo* observation.

### 3.2 Immunofluorescence technique

The immunofluorescence technique utilizes fluorescently labeled antibodies as probes to detect the expression of specific antigens within liver tissue or cells, followed by scanning and imaging using fluorescence microscopy or confocal imaging. This method enables us to observe the localization of HSCs, hepatocytes, bile duct epithelial cells, KCs, LSECs, or proteins and to understand the patterns of cellular morphological changes (He et al., 2023). Immunofluorescence technology offers the advantage of detecting expression of target molecules with strong specificity and achieving multi-color fluorescence staining to detect the position and interrelationships of target molecules. However, it typically cannot be carried out directly in living animals or cells, and fluorescent dyes may gradually quench due to prolonged exposure, thus limiting observation time and continuity.

### 3.3 Fluorescence *in situ* hybridization (FISH)

FISH utilizes the principle of complementary base pairing to hybridize fluorescently labeled probes with target DNA following denaturation and renaturation, enabling direct visualization of the target DNA’s location using fluorescence microscopy, confocal microscopy or other equipment. The location of the periportal vein regions, central vein regions, or cells and the status of hepatic sinusoid damage (Ben-Moshe et al., 2022) could be observed by using FISH to label specific target genes in hepatic microcirculation. FISH offers high resolution and strong specificity, achieving visualization of the distribution and expression of target genes. However, this method entails complex sample processing to maintain tissue structural integrity and stability. Additionally, it may lack sufficient sensitivity for detecting genes with low expression levels.

### 3.4 Scanning electron microscope (SEM)

While H&E staining provides clear insight into the macroscopic morphology and structure of the liver, it falls short of revealing its nanoscale structure. To address these limitations, researchers utilize SEM to observe the liver microstructure. SEM operates on the principle of using electron beams to scan the sample’s surface, generating high-resolution images. Within liver tissue, SEM enables the observation of various microstructures, including hepatocytes, endothelial cells, KCs, leukocytes, fenestrae, filopodia, and collagen fibers. These observations could directly reflect microscopic spatial morphological changes within the liver (Nafady et al., 2017). However, it is essential to note that SEM imposes stringent requirements on sample preparation. Damage to tissue samples must be meticulously avoided during fixation, dehydration, drying, gold-plating, and other processes, as any such damage can significantly impact microstructure observations. Additionally, SEM is restricted to capturing two-dimensional surface information of the sample and cannot provide a clear view of organelle structures or three-dimensional arrangements.

### 3.5 Transmission electron microscopy (TEM)

It is essential to explore the changes in subcellular structures within cells. TEM employs electron beams to penetrate samples, generating high-resolution, high-contrast images. It allows for a detailed examination of the ultra-microstructure of the liver, including mitochondria, cell nuclei, rough endoplasmic reticulum, microvilli, Disse space, lysosomes, vesicles, and collagen deposition (Nafady et al., 2017). Nonetheless, TEM presents challenges in sample preparation, risking potential damage. Prepared samples must endure vacuum conditions and exposure to high-energy electron beams. Additionally, TEM is limited to observing thin tissue sections, lacking the ability to detect the tissue’s three-dimensional structure.

### 3.6 Color Doppler ultrasound

Color Doppler ultrasound is a better way to visualize the shape and function of blood vessels and blood flow characteristics (Zhang and Han, 2021). The principle of color Doppler ultrasound relies on the Doppler effect, which means that when ultrasound encounters moving erythrocytes, the frequency will change, reflecting the direction, speed, and distribution of blood flow. Color Doppler ultrasound codes blood flow in different directions and speeds with different colors. Then, it superimposes them on the two-dimensional image to form a color Doppler ultrasound blood flow image. This technique could help to identify vessels, assesses blood flow as well as detect the hemodynamic characteristics of the hepatic artery, portal vein, and hepatic vein (Tanaka, 2020). It can visually display the vascular structure and hemodynamic characteristics in liver, which is of great value in diagnosing liver lesions. However, color Doppler ultrasound still possesses certain limitations in detecting deep tissues.

### 3.7 Laser speckle imaging system

Researchers have developed a laser speckle imaging system with a higher spatial resolution to capture more detailed and comprehensive blood flow information (Li et al., 2021). Exposing tissue containing flowing blood cells to laser irradiation generates random interference speckle patterns, which underlie the principle behind this technology. These speckle patterns change with variations in blood flow. Analyzing temporal and spatial changes in speckle patterns enables the acquisition of information on blood flow velocity and distribution. This breakthrough makes it possible to visualize blood flow conditions in real-time, particularly in liver diseases like NAFLD (Pan et al., 2021). Remarkably, however, the laser speckle imaging system is limited to detecting blood flow at a depth of 1 mm below the tissue surface.

### 3.8 Two-photon imaging technology

Whereas the laser speckle imaging system offers benefits like non-contact, non-invasive, and rapid imaging, it suffers from low resolution in deep tissues, making it challenging to distinguish individual blood vessels. In contrast, two-photon imaging employs two low-energy infrared photons to achieve deep three-dimensional imaging in living tissues, with depths of up to 250–500 μM or exceeding 1 mm. Researchers can utilize this instrument to observe the inner diameter of liver sinusoids and calculate the blood flow velocity in the sinusoids based on distance-time images (Fan et al., 2019). An advantage lies in using near-infrared light, which causes less damage to biological tissues, allowing for long-term, high-resolution functional imaging of living tissues. Nevertheless, this technology presents challenges, including demanding design requirements for fluorescent probes, high equipment costs, and the need for objective quantitative analysis methods and standardized evaluation criteria, which require further research and validation to address these limitations.

### 3.9 Super microvascular imaging (SMI)

SMI technology is a cutting-edge hepatic microcirculation detection method with the advantages of non-invasive, radiation-free and no contrast agent requirement. It employs intelligent filtering to isolate very low-speed (min. 0.8 cm/s) blood flow signals from tiny vessels (diameter >0.1 mm) in liver lesions. This technology can help to identify benign/malignant liver lesions, assess tumor metastasis, and gauge liver function and perfusion (He et al., 2017). However, the main drawback of SMI is its limit ability to quantify blood flow velocity, which cannot provide parameters such as blood flow velocity, direction, and resistance index. There is still a need for clinical validation and standardized assessment methods. Further research and verification are required to establish the diagnostic efficacy and clinical significance.

### 3.10 Single-cell sequencing

Single-cell sequencing is a genomics approach that relies on comprehensive, high-throughput genomic analysis of individual cells through RNA amplification technology. It detects gene expression patterns and transcriptome features of individual cells, thereby annotating multiple cell types and providing insight into the diversity of cellular states. For example, researchers achieved to obtain transcriptional profiles of 20 discrete cell populations, including hepatocytes, endothelial cells, cholangiocytes, HSCs, B-cells, T-cells and NK cells by single-cell sequencing. This comprehensive analysis delineates the characteristics of the resident cells in the liver and provides a detailed map of the immune microenvironment of the human liver (MacParland et al., 2018). Analyzing the gene expression of different cell types in the hepatic sinusoidal microcirculation enables the identification of changes in the expression patterns of specific genes and cellular subpopulations associated with abnormal microcirculatory function. This capability aids in uncovering the underlying biological mechanisms of microcirculatory disturbance, providing an essential basis for diagnosing and treating related diseases. Despite the advancements in single-cell sequencing technology, challenges persist in processing and analyzing large datasets. Furthermore, it still needed to elucidate global transcriptional differences across lobular units in physical space.

### 3.11 Space transcriptomics

Spatial transcriptomics is a genomic approach based on detecting gene expression and spatial location information on tissue sections through the combination of spatial localization technology and high-throughput RNA sequencing to reveal the distribution of cell types in tissues and the interactions between adjacent cells. Researchers have conducted studies to spatially annotate the hepatic sinusoids at the level of the liver lobules using spatial transcriptomics to classify the hepatic sinusoids into periportal, intermediate, and pericentral venous zones and to compare the overall transcriptional differences in the hepatic lobular axis (Hildebrandt et al., 2021). This approach can provide a crucial spatial analysis tool for revealing the pathophysiological mechanisms of microcirculatory disturbance and help to develop precise therapeutic strategies for related diseases. However, it still needs to be continuously improved in sample processing and imaging resolution to enhance the efficiency and accuracy of its application in the study of hepatic sinusoidal microcirculation.

Beyond these methods, various approaches can be used for observing and evaluating hepatic microcirculation, such as immunoelectron microscopy (Yokomori et al., 2015), perfusion-weighted MRI (Ding et al., 2021), computed tomography (CT) angiography (Kim J. S. et al., 2022), hepatic arteriography (Murata et al., 2014), inverted intravital microscope (Mu et al., 2018), laser Doppler flowmetry (Papagiouvanni et al., 2022), CT perfusion imaging (Brehmer et al., 2018), contrast-enhanced Ultrasound (CEUS) (Pang et al., 2018) (Table 1). Each has unique advantages and disadvantages. In practice, the choice of detection method should align with specific needs to obtain precise and comprehensive information about hepatic microcirculation.

**TABLE 1:** Commonly used methods for the detection of hepatic microcirculation.

Method	Observable liver structures	Principle	Advantages	Disadvantages	Reference
H&E staining	Hepatocytes, central vein, portal vein, hepatic artery, and bile duct, etc	Using hematoxylin and eosin to label the basophilic and eosinophilic structures of liver tissue	Easy to operate and to diagnose diseases	Unable to provide molecular functional information inside cells; Nor distinguish between proteins, nucleic acids, or polysaccharides within tissues; Only capable of providing two-dimensional information and unable to conduct *in vivo* observations	Boyd et al. (2020)
Immunofluorescence	Localization of HSCs, hepatocytes, bile duct epithelial cells, KCs, LSECs, or proteins, and to understand the patterns and quantities of cellular morphological changes	Using fluorescently labeled antibodies as probes to detect the expression of specific antigens within liver tissue or cells	High detection sensitivity; Strong specificity; Multicolor fluorescence staining	Cannot be performed in living organisms; Fluorescent dyes may fade over time	He et al. (2023)
Fluorescence *in situ* hybridization	Location of the periportal vein regions, central vein regions or cells, and the status of hepatic sinusoid damage	Using complementary base pairing to hybridize fluorescent probes with target DNA after denaturation and renaturation, allowing direct visualization of the target DNA with fluorescence microscopy	High resolution; High specificity; Visualization of target gene distribution and expression	Complex sample processing; Insufficient sensitivity for low-expression genes	Ben-Moshe et al. (2022)
Scanning electron microscope	Hepatocytes, endothelial cells, KCs, leukocytes, sinusoids, pseudopodia, collagen fibers, etc	Generating high-resolution images by scanning a focused electron beam across the sample surface and detecting various interactions	Providing high-resolution microscopic structural information	Requires complex sample preparation; Unable to provide three-dimensional information. Unable to conduct *in vivo* observation	Nafady et al. (2017)
Transmission electron microscopy	Mitochondria, cell nucleus, endoplasmic reticulum, microvilli, Disse space, lysosomes, vesicles, collagen deposition, etc	Using an electron beam to penetrate the sample and generate high-resolution images	Providing detailed internal structural information	High sample preparation requirements; Cannot observe thick tissue slices or three-dimensional structures; Unable to proceed *in vivo* observation	Nafady et al. (2017)
Immunoelectron microscopy	Vascular endothelial cells, LSECs, HSCs, hepatocytes, fenestrae structure or protein localization	Gold-labeled secondary antibodies are bound to the primary antibodies, and finally, the electron microscope is used to locate the target antigen	Observable protein localization in liver microstructure; High-resolution	Complex sample preparation; High cost; Requires advanced technical skills; Susceptible to electron beam damage during operation	Yokomori et al. (2015)
Laser speckle imaging system	Detect blood flows at a depth of 1 mm below the tissue surface	When the target is illuminated by a laser beam, the reflected laser forms a random interference pattern	Non-contact, non-invasive, rapid imaging	Limited to a depth of 1 mm	Pan et al. (2021)
Inverted intravital microscope	Detect the speed and direction of blood flow in liver microvasculature and the number of hepatic sinusoids	Using a high-resolution microscope and appropriate fluorescent markers to observe the liver vascular structure and blood flow dynamics via laser, white light, or tissue surface exposure	Real-time observation; High-resolution	Complex operations, high costs; High requirements for professional skills	Mu et al. (2018)
Two-photon imaging technology	Detect hepatic sinusoidal diameter and blood flow velocity	Using two low-energy infrared photons to achieve deep three-dimensional imaging, providing high resolution and depth information	Deep penetration; High resolution	High equipment costs; Requiring specific fluorescent probes	Fan et al. (2019)
Super microvascular imaging	Visualize slow blood flow in tiny blood vessels	Using super microvascular imaging technology to detect tiny blood vessels	Non-invasive; Radiation-free; Contrast-agent-free; Suitable for small blood vessels	Further clinical validation and standard assessment methods are required	He et al. (2017)
Color Doppler ultrasound	Detect the blood flow velocity, direction, and vascular morphology of the hepatic artery, portal vein, and hepatic vein	Combines the information from pulsed wave Doppler with color coding to show the direction of blood flow	Non-invasive, intuitive display of vascular structure and blood flow	Limited detection depth	Tanaka (2020)
Laser Doppler Flowmetry	Detection of liver blood flow velocity and volume	Based on the laser Doppler effect. When the laser irradiates moving red blood cells, the frequency of the light changes. This frequency shift is used to calculate the blood flow velocity and volume	No need for puncture procedures; Real-time acquisition of blood flow data	Can only measure surface tissues; Accuracy affected by the light scattering properties of the tissue	Papagiouvanni et al. (2022)
Perfusion-weighted MRI	Detect the perfusion status of the liver	Using a specific MRI sequence to monitor liver hemodynamic parameters like blood flow and volume	No need for intubation or injection of toxic substances; High spatial resolution; Dynamic observation	Requires contrast agent; Higher cost; Longer detection time	Ding et al. (2021)
Contrast-enhanced Ultrasound	Blood vessel structure; blood perfusion	Interaction between ultrasound and microbubble contrast agents	No radiation; Real-time dynamic observation; Contrast agents of CEUS metabolize and excrete faster than those in MRI	Only suitable for superficial organ and lesion assessment; Limited penetration of ultrasound	Pang et al. (2018)
CT angiography	Visualization of blood vessels and assessment of vascular structures throughout the liver	Based on the X-ray absorption characteristics and the contrast enhancement effect of iodine-based contrast agents	High-resolution; Non-invasive detection method	Radiation exposure; Iodine-based contrast agents may impact renal function; Inability to observe hemodynamics in real-time	Kim et al. (2022b)
CT Perfusion Imaging	Liver hemodynamics and blood supply	Based on dynamically tracking the distribution and washout of contrast agents in the liver	Captures dynamic changes in blood flow in real-time; Non-invasive technique	High radiation dose	Brehmer et al. (2018)
Hepatic arteriography	Visualize the structure of the hepatic artery and its branches; Assess blood flow dynamics; Highly sensitive to the abnormal vascular proliferation associated with liver tumors	Using X-ray imaging to observe vascular and hepatic images by injecting contrast agent into the hepatic artery system	High resolution and sensitivity make it suitable for guiding hepatic artery embolization therapy	Requires catheter insertion; Radiation exposure; Primarily focuses on the hepatic artery system, with limitations in evaluating other pathologies	Murata et al. (2014)
Single-cell sequencing	Gene expression patterns of different cell types such as hepatocytes, HSCs, hepatic sinusoidal endothelial cells, and koilocytes, as well as their subcellular populations	Comprehensive high-throughput genomic analysis of individual cells by RNA amplification technology	With the ability to discover cell type, subtype and transcriptome heterogeneity	Large amount of processed data and high complexity of analysis	MacParland et al. (2018)
Space transcriptomics	Spatial distribution of cell types such as hepatocytes, HSCs, hepatic sinusoidal endothelial cells, etc	Combining spatial localization technology with high-throughput RNA sequencing to detect gene expression and spatial location information on tissue sections, revealing cell type distribution and interactions between neighboring cells	Preserve tissue structure information and provide spatial distribution of gene expression	Complex sample processing steps; Low imaging resolution	Hildebrandt et al. (2021)

## 4 Microcirculatory disturbance in liver diseases

The liver’s normal function relies on the integrity and stability of hepatic microcirculation. Nonetheless, various factors such as viral infections, alcohol consumption, hypoxia, and ischemia can disrupt hepatic microcirculation, leading to conditions such as acute liver failure, viral hepatitis, alcoholic liver disease, and hepatocellular carcinoma. This section will summarize the characteristics of microcirculatory disturbance in various liver diseases.

### 4.1 Acute liver failure (ALF)

ALF refers to a large amount of hepatocyte necrosis, apoptosis, and severe liver dysfunction occurring within a short period in the absence of underlying liver disease, characterized by severe coagulation dysfunction and encephalopathy. Liver transplantation is the ultimate curative option for ALF. Although in some cases, spontaneous regeneration is possible if the patient is managed conservatively in intensive care units (Ribaud et al., 2022). Microcirculatory disturbance in ALF manifests through various abnormalities, including HSCs and KCs activation, fibrin deposition, and thrombosis (Figure 2A). Firstly, study has elucidated the pivotal role of HSCs in ALF, in which HSCs relays inflammation signaling from sinusoids to parenchyma via the secretion of inflammatory cytokines. Conversely, HSCs aid in liver regeneration by releasing growth factors and maintaining hepatocyte attachment and liver tissue architecture via ECM production (Li et al., 2019). Additionally, study also have revealed that activated KCs contribute to ALF by secreting chemokines that recruit monocytes, neutrophils, and cytokines, thus exacerbating inflammation and sensitize hepatocytes to apoptosis. Neutrophils and monocyte-derived macrophages also could secrete cytokines and pro-angiogenic factors (Krenkel et al., 2014; Kolodziejczyk et al., 2020). In ALF, damage to LSECs, characterized by degeneration, necrosis, and detachment, triggers fibrin deposition in liver sinusoids and platelet aggregation, contributing to intravascular coagulation (Hirata et al., 1989).

### 4.2 Hepatic ischemia-reperfusion injury (HIRI)

HIRI is a pathological condition characterized by hepatocyte damage and inflammation triggered by transient ischemia and subsequent restoration of blood flow, commonly occurring during surgical procedures or transplantation (Nakamura et al., 2019). Recent investigation has demonstrated that the administration of prostaglandin (PG)E1 can ameliorate microcirculation dysfunction in hepatic I/R syndrome by expanding blood vessels and enhancing perfusion status (Mouratidou et al., 2023). The primary manifestations of hepatic microcirculatory disturbance in HIRI encompass abnormal hemodynamics, hepatocyte necrosis and apoptosis, activation of KCs and HSCs (Figure 2B). During HIRI, notable sinusoidal congestion occurs, accompanied by edema, deformation, necrosis and apoptosis of KCs, LSECs and hepatocytes. Additionally, hepatocytes may display cytoplasmic vacuolization, while LSECs may exhibit plasma membrane rupture, nuclear membrane vacuolization and cell morphological changes, collectively contributing to the narrowing of sinusoidal space (Peralta et al., 2013; Sheng et al., 2015; Mu et al., 2018). In terms of inflammation, upregulation of nuclear factor-κB (NF-κB) p65 acetylation occurs, along with KCs and neutrophils releasing inflammatory mediators and ROS. This cascade promotes neutrophil recruitment and adhesion to sinusoids, disrupting LSECs and microvascular integrity (Peralta et al., 2013; Tao et al., 2014; Cannistrà et al., 2016). Activated HSCs release cytokines, rho-associated kinase, endothelin-1 (ET-1) and matrix metalloproteinases (MMPs), thereby stimulating the recruitment of CD4^+^ T cells to the injury site and increasing the quantity and activity of inflammatory factors (Peng et al., 2022). Under the stimulation of pro-inflammatory cytokines, HSCs are activated and differentiate into myofibroblasts, thereby inducing fibrosis through ECM deposition (Liu et al., 2019). Researchers have observed a significantly reduction in the number and proportion of hepatocytes following HIRI injury, with the pericentral venous zone exhibiting heightened sensitive to HIRI injury by using spatial transcriptomics. Furthermore, enrichment of endothelial cells, epithelial cells and HSCs was observed in the periportal zone, revealing pericentral zone-specific injury-related change in differentially expressed genes, cellular composition and functional pathways following HIRI injury (Xin et al., 2023).

### 4.3 Viral hepatitis

Viral hepatitis refers to liver inflammation caused by different types of viruses, such as hepatitis A virus (HAV), hepatitis B virus (HBV) and hepatitis C virus (HCV). Initially, interferon therapy was utilized following the identification of viral hepatitis. However, due to its limited efficacy and significant side effects, treatment strategies later transitioned towards antiviral medications (Bush et al., 2023). The pathogenesis of hepatic microcirculatory disturbance in viral hepatitis involves coagulation dysfunction, capillarization and HSCs activation (Figure 2C). Studies has found that HBV and HCV primarily attack hepatocytes, where they replicate and release extensively (Maini and Peppa, 2013; Klenerman and Ramamurthy, 2015). HBV infection can cause narrowing of sinusoidal space and the decreased of blood flow (Maini and Peppa, 2013). In advanced stages of the HBV infection, HSCs activation is induced through endoplasmic reticulum stress and ferroptosis pathways, ultimately leading to fibrosis (Kuo et al., 2020). Similarly, During HCV infection, elevated thrombin levels, along with the presence of numerous thrombi in the portal vein, lead to HSCs activation and hepatic fibrosis progression (González-Reimers et al., 2016). In the early stages of infection with mouse hepatitis virus type 3, microthrombi form occurs in the portal vein and periportal sinusoids, resulting in obstruction of sinusoidal blood flow. Subsequently, during the mid-stage of viral infection, hepatocyte swelling ensues, causing altered blood flow patterns from damaged to undamaged areas. Finally, in the late stage of viral infection, extensive thrombus formation and hepatocyte death occur (Levy et al., 1983).

### 4.4 Non-alcoholic fatty liver disease (NAFLD)

NAFLD is characterized by the excessive accumulation of fat in the liver, in the absence of alcohol abuse or other clear causes. Despite its prevalence, there are currently no approved pharmacological interventions for NAFLD (Pereira et al., 2022). Notably, simvastatin has shown to improve microcirculatory function in NAFLD by mitigating oxidative and advanced lipoxidation end product–receptors of advanced glycation end products (ALE-RAGE) stress, while also ameliorating steatosis, fibrosis and inflammatory markers (Pereira et al., 2022). Microcirculatory disturbances in NAFLD are typified by the activation of HSCs and KCs, along with the narrowing of sinusoidal space and diminished blood flow (Figure 2D). Studies have indicated that a high-fat diet can lead to structural and functional alterations in LSECs, activation of HSCs, enhanced release of NO by KCs, as well as increased adhesion of macrophages and monocytes within the hepatic sinusoids, exacerbating oxidative stress. Additionally, it induces hepatocyte enlargement and deposition of ECM into the Disse space, thereby inducing liver microcirculatory damage and a 42% reduction in hepatic blood flow (Pereira et al., 2017; Nasiri-Ansari et al., 2022). Moreover, excessive triglycerides (TG) accumulation can also induce swelling and apoptosis of hepatocyte, and platelet aggregation, further contributing to the narrowing of sinusoidal space, and reduced blood flow (Fan et al., 2019; Miao et al., 2024). High-fat diets also elevate liver vascular tension and perfusion while impairing endothelial dilation response to acetylcholine (Ach), thereby disrupting normal blood flow dynamics (Pasarín et al., 2012). As NAFLD progresses, disappearance of LSECs fenestrae, formation of the basement membrane forms and extensive ECM deposition occurs in the Disse space, ultimately leading to fibrosis (Baffy, 2018).

### 4.5 Alcoholic liver disease (ALD)

ALD is a liver disorder caused by prolonged and excessive alcohol consumption. While modest alcohol intake can appropriately increase liver blood flow, acute alcohol consumption can cause hepatic microvascular dysfunction, exacerbating gut I/R-induced hepatic microvascular dysfunction and subsequent liver injury (Israel and Orrego, 1987; Horie and Ishii, 2001). Although glucocorticoids and hepatic protectants have received FDA approval for ALD treatment, ongoing debate surrounds their efficacy (Mai et al., 2022). Microcirculatory disturbance in ALD is associated with sinusoidal capillarization, and inflammation (Figure 2E). Researchers have found that hepatocyte enlargement in ALD leads to sinusoidal vessel compression, disrupting erythrocyte circulation within liver sinusoids and impeding hepatic microcirculation (Mak et al., 2022). Ethanol consumption can also modulate the composition of the intestinal microbiota, facilitating the translocation of gut-derived lipopolysaccharide (LPS) and other bacterial products into the portal vein. Subsequent activation of toll-like receptor (TLR) 4 signaling in KCs incites liver inflammation (Inokuchi et al., 2011). Moreover, ethanol intake reduces the number and diameter of fenestrations in LSECs, induces marginal contraction of LSECs, and activates HSCs, leading to increased synthesis of collagen fiber bundles and resultant sinusoidal capillarization (Mak et al., 2022).

### 4.6 Hepatic fibrosis and cirrhosis

Hepatic fibrosis represents an abnormal wound healing response in the liver caused by long-term liver damage from diverse etiologies. Currently, drugs for treating hepatic fibrosis exit limited efficacy and lack clinical and commercial validation. Although several medications, including Selonsertib, Simtuzumab, and GR-MD-02, are undergoing clinical trials for hepatic fibrosis treatment, none have garnered approval (Zhang et al., 2024). Microcirculatory disturbance in hepatic fibrosis mainly manifests as increased immune response, HSCs activation, capillarization and reduced blood flow (Figure 2F). In fibrotic conditions, hepatocytes undergo ballooning degeneration accompanied by degenerative necrosis (Fu et al., 2021). Neutrophils play a pivotal role in the activation of HSCs by generating ROS and myeloperoxidase (MPO). Subsequently, activated HSCs, in turn, secrete cytokines such as granulocyte-macrophage colony-stimulating factor (GM-CSF) and IL-15, thereby promoting neutrophil activation. Additionally, activated HSCs release neutrophil chemotactic factors, recruiting more neutrophils, forming a positive feedback loop that promotes the development of hepatic fibrosis (Tang et al., 2021). KCs contribute to the activation of HSCs and the survival of myofibroblasts by secrete TGF-β1 and PDGF (Krenkel and Tacke, 2017). KCs also release inflammatory factors like IL-1β and chemokines which activate HSCs and recruit other immune cells (Roehlen et al., 2020). An *in vitro* study indicates that LPS can activate liver TLR4 signaling pathway, transform hematopoietic stem cells into fibroblasts, and produce inflammatory factors like NF-κB to activate HSCs (Liang et al., 2016). Additionally, multiple signaling pathways, including Src/extracellular regulated protein kinases/*drosophila* mothers against decapentaplegic homolog 3 (Src/ERK/Smad3) (Huang Y. et al., 2023), Sirt1/Notch (Sun et al., 2022), ferroptosis (Huang et al., 2022), and phosphatase and tensin homolog (Geng et al., 2020) are all involved in the activation of HSCs and hepatic fibrosis. Deposition of collagen and other ECM proteins culminates basement membrane and loss of fenestrae, resulting in the capillarization of LSECs (Lafoz et al., 2020; Ma et al., 2021). Imbalances in vasoactive substances like ET-1, AngⅡ, norepinephrine, NO, carbon monoxide, thromboxane A2 (TXA2), PGI2, etc., increase intrahepatic resistance, thereby reducing hepatic sinusoidal blood flow (Huang Q. et al., 2023; Fu et al., 2023; Velez et al., 2024). Furthermore, VEGF and its receptors VEGFR1 and VEGFR2 promote abnormal angiogenesis in hepatic fibrosis (Wang et al., 2021).

Cirrhosis is the later stage of fibrosis. It characterized by portal hypertension leading to gastroesophageal varices and bleeding , which is the primary cause of mortality in cirrhotic patients (Gunarathne et al., 2020). Currently, effective treatments for preventing cirrhosis are lacking, thus the focus is primarily on managing liver diseases and their associated complications (Mendez-Guerrero et al., 2024). Microcirculatory disturbance in hepatic cirrhosis manifest through various abnormalities including abnormal neovascular proliferation, vascular occlusion, HSCs activation, capillarization and thrombosis (Figure 2F). Studies have shown an increase in the number of abnormal hepatic sinusoidal vessels in cirrhotic conditions (Thabut and Shah, 2010). Elevated coagulation factor VIII and decreased anticoagulation factor protein C result in portal and hepatic vein occlusion (Tripodi, 2015). Furthermore, activated KCs in the liver secrete TGF-β and inflammatory factors, contributing to reduced fenestrae in LSECs (Yang et al., 2009). Impaired LSECs function also affects substance exchange within liver sinusoids, leading to increased secretion of vasoconstrictive factors like ET-1, Ang II, PGH2, TXA2, and decreased secretion of vasodilatory factor NO. This imbalance induces pericellular contraction and thrombus formation, subsequently elevated intrahepatic pressure (Bosch et al., 2015; Gracia-Sancho et al., 2019; Gunarathne et al., 2020). Moreover, aberrant LSECs’ function reduces sinusoidal permeability, leading to hepatic ischemia, which further stimulates HSCs to secrete collagen, thereby exacerbating fibrosis (Thabut and Shah, 2010; Greuter and Shah, 2016; McConnell and Iwakiri, 2018). Recent advancements utilizing single-cell sequencing have identified a novel subpopulation of scar-associated TREM2^+^CD9^+^ macrophages with pro-fibrotic effects in cirrhotic disease. Additionally, researchers have defined novel ACKR1^+^ and PLVAP^+^ endothelial cells that expand in cirrhosis, are topographically scar-restricted, and enhance leucocyte transmigration (Ramachandran et al., 2019).

### 4.7 Hepatocellular carcinoma (HCC)

HCC primarily evolves from chronic viral hepatitis, cirrhosis, prolonged alcohol abuse and other liver diseases. Mainstream medicine employs various strategies for HCC treatment including surgical resection, liver transplantation, radiofrequency, chemotherapy and targeted molecular therapy (Kim et al., 2022). Microcirculatory disturbance in HCC involves not only capillarization and activation of KCs but also neovascularization and the formation of platelet-tumor cell aggregates (Figure 2G). In the development of HCC, tumor suppressor mechanisms are inhibited, leading to an increased risk of carcinogenesis via DNA mutations. Chronic hepatitis-induced hepatic sinusoidal inflammation and ROS contribute to increased DNA damage and proliferation of cancer cells (Gibert-Ramos et al., 2021). During the cirrhotic stage, the accumulation of matrix cells promotes the proliferation of HCC cells (Baglieri et al., 2019). Simultaneously, HSCs produce growth differentiation factor 15 (GDF15) through autophagy-dependent pathways, further promoting the proliferation of HCC cells (Myojin et al., 2021). Extensive fibrosis and capillarization inducing hypoxia in liver sinusoids, enable KCs to release various chemokines and cytokines. Through the HIF-1 pathway, they can secrete PDGF-β, VEGF, angiopoietin-1 (ANG-1), and MCP-1, thereby impairing the function of CD8^+^ T cells and dampening their anti-tumor effects, ultimately promoting tumor growth and metastasis (Liu et al., 2011; Gibert-Ramos et al., 2021). As the tumor progresses, tumor and surrounding cells secrete VEGF, fibroblast growth factor (FGF), PDGF and other angiogenic factors. These factors stimulate the proliferation, migration and differentiation of endothelial cell, thereby forming new capillaries and creating a microenvironment favorable for tumor cell proliferation (Li, 2016). Midkine (MK) can modulate NF-κB and promote the expression of VEGF, Ang-2, and Tie2 by activating integrin α4 (Itgα4) through autocrine signaling, inducing pathological angiogenesis (Fu et al., 2023). Tumor cells shed from the primary focus into the bloodstream can induce platelets to aggregate on their surfaces, forming platelet-tumor cell aggregates. This process helps tumor cells evade immune system attacks and shear force damage (Kanikarla Marie et al., 2021). Simultaneously, platelet aggregates can carry tumor cells to other organs, facilitating their adhesion to blood vessel walls and promoting tumor metastasis (Kanikarla Marie et al., 2021).

## 5 Potential effects of TCM on hepatic microcirculatory disturbance

TCM formulas and their active metabolites play potential roles in protecting liver cells and promoting repair and regeneration. They possess the ability to regulate qi and blood, invigorate blood circulation, and remove stasis. These properties have demonstrated significant efficacy in managing hepatic microcirculatory disturbance by increasing blood flow, reducing thrombosis, and inhibiting abnormal angiogenesis and capillarization. Consequently, TCM formulas have exhibited remarkable therapeutic effects in various liver diseases such as ALF, HIRI, NAFLD, hepatic fibrosis, and HCC.

Research has demonstrated the potential of TCM formulas and their active metabolites in regulating hepatic microcirculatory disturbance by reducing thrombosis and increasing blood flow. For example, Shen-Ling-Bai-Zhu-San, a formula traditionally used to tonify the spleen and eliminate dampness, has been shown to improve organelle morphology, reduce hepatocyte necrosis, and alleviate lipid droplet accumulation in hepatocytes of NAFLD. Additionally, liver perfusion measured by a moorFLPI-2 blood flow imager showed significant improvement, possibly regulated by serum adiponectin (Tang et al., 2020; Pan et al., 2021). Active metabolites also play a significant role to these effects. Caffeic acid 1), extracted from *Salvia miltiorrhiza* Bge. [Lamiaceae; Salviae miltiorrhizae radix et rhizoma], was found to restore hepatic sinusoidal perfusion and erythrocyte velocity in HIRI mice using an inverted intravital microscope and Laser-Doppler Perfusion Imager. It was also reported to reduce leukocyte adhesion, blood cell count, liver lobule distortion, hepatic sinusoidal disturbance, congestion, hepatocellular vacuolization, and necrosis (Mu et al., 2015; Mu et al., 2018). Similarly, plumbagin 2), an active metabolite of *Plumbago zeylanica* L. [Plumbaginaceae; *Plumbago* radix et folium], has shown promise in reducing liver thrombosis, inflammatory cell infiltration, and macrophage recruitment in mice with ALF (Wang et al., 2016). Berberine 3) from *Coptis chinensis* Franch. [Ranunculaceae; Coptidis rhizoma] effectively alleviated HIRI symptoms such as hepatic lobular edema, hemorrhage, deformation and necrosis, inhibited neutrophil inhibited neutrophil infiltration and hepatocyte apoptosis (Sheng et al., 2015). Furthermore, acteoside 4), extracted from *Lantana camara* L. [Verbenaceae; Lantana radix, folium and flos], was reported to reverse the senescent fate of LSECs, restore sinusoidal networks, as well as ameliorate sinusoidal congestion, vacuolization, hepatocyte necrosis and oxidative stress by targeting the HMGB1-TLR3/4-IRF1 signaling pathway, thus providing protection against HIRI and offering the potential for new therapeutic developments (Jia et al., 2023).

Furthermore, TCM formulas and their active metabolites offer potential in improving hepatic microcirculatory disturbance by regulating abnormal angiogenesis. For instance, formulas like Si-Ni-San, aimed at soothing the liver and resolving stagnation, have been shown to inhibit angiogenesis in hepatic fibrosis tissues, reverse the activation of HSCs, reduce ECM accumulation, and alleviate hepatic fibrosis (Wang et al., 2021). Similarly, Xia-Yu-Xue decoction exerts its anti-angiogenic effects by decreasing the activities of MMPs (MMP-2 and MMP-9), inhibiting HSC activation, and damaging the integrity of new vessels, thus improving hepatic fibrosis (Du et al., 2011). Moreover, Xue-Fu-Zhu-Yu decoction demonstrates inhibitory effects on angiogenesis, hypoxia alleviation, and protective effect on LSECs function, thereby improving hepatic fibrosis (Zhou et al., 2014). The Tao Ren-Hong Hua herb pair can inhibit pathological hepatic angiogenesis, inflammation and fibrosis induced by carbon tetrachloride (CCL4) in chronic liver disease (Xi et al., 2016). Da-Huang-Zhe-Chong pill, which focuses on dispelling pathogenic factors, breaking blood stasis, and promoting blood circulation, can reduce pathological angiogenesis in HCC by inhibiting the MK/Itgα4 signaling pathway (Fu et al., 2023). Yi-Guan-Jian decoction can inhibit liver angiogenesis in cirrhotic mice treated with CCl4 by inhibiting the HIF-1α/VEGF signaling pathway (Zhou et al., 2015). Furthermore, gene Ontology analysis found that Jie-Du-Hua-Yu granule protects against liver failure by negatively regulating angiogenesis, fibrinolysis, and cell shape (Qiu et al., 2019). Yu-Ping-Feng-San attenuates the activation of the thymic stromal lymphopoietin-signal transducer and activator of transcription 3 (TSLP-STAT3) signaling pathway by inhibiting the immune-related factor TSLP, thereby inhibiting the formation of hepatic microvessels and exerting an anti-HCC effect (Yuan et al., 2019). Jie-du recipe may inhibit hypoxia-induced angiogenesis by suppressing IL-8/HIF-1α/phosphatidylinositol-3-kinase (P13k) and mitogen-activated protein kinase (MAPK)/ERK pathways after transcatheter arterial chemoembolization in HCC patients (Lin et al., 2021). Active metabolites such as Amarogentin 5), extracted from *Swertia davidii* Franch. [Gentianaceae; Swertia davidii Franch herba] can inhibit cancer cell angiogenesis by affecting stemness and the p53-dependent VEGFA/Dll4/Notch1 signaling pathway, thus preventing the malignant transformation of liver cancer cells (Zhang et al., 2020). Levistilide A 6), an active metabolite of *Angelica sinensis* (Oliv.) Diels. [Apiaceae; Angelicae sinensis radix], can inhibit hepatic fibrosis through anti-angiogenesis by alleviating sinusoid capillarization via the VEGF signaling pathway (Zhao et al., 2017). Hydroxysafflor Yellow A 7) from *Carthamus tinctorius* L. [Asteraceae; Carthami flos] has the potential to significantly suppress tumor growth by inhibiting the secretion of angiogenesis factors, such as VEGF-A and basic FGF, as well as VEGFR1 (Yang et al., 2015). Additionally, it can also suppress angiogenesis in HCC by regulating the p38 MAPK signaling pathway (Zhang et al., 2019). Oroxylin A 8), an active metabolite of *Cutellaria baicalensis* Georgi. [Lamiaceae; Scutellariae baicalensis radix], can inhibit hypoxia-induced nuclear translocation of YAP, which may influence the accumulation of HIF-1α and subsequently decrease the transcription of downstream target genes, including VEGF-A and Ang-2, thereby exerting anti-angiogenic activity (Zhang et al., 2018).

In addition, certain active metabolites have been reported to regulate hepatic microcirculatory disturbance by addressing sinusoidal capillarization. For instance, Curcumol 9), an extract of *Curcuma longa* L. [Zingiberaceae; Curcumae longae rhizoma], has shown to restore microcirculation and improve sinusoidal capillarization in hepatic fibrosis (Zheng et al., 2020).

In summary, TCM formulas (Table 2) and their active metabolites (Table 3; Figure 3) demonstrate promise to improve hepatic microcirculation in diverse liver diseases. They maintain the stable structure of the liver antrum and enhance blood circulation by promoting blood flow and removing blood stasis, thus preventing diseases such as fibrosis, cirrhosis, and HCC. As safe and effective drugs, TCM offers a valuable adjunctive treatment option for patients with liver diseases.

**TABLE 2:** Chinese medicinal formulae with potential activity in regulating hepatic microcirculatory disturbance.

TCM formula or TCM	Composition	Disease	Mouse/patient	Therapeutic effects	Reference
Shen-Ling-Bai-Zhu-San	*Dolichos lablab* L. [Fabaceae; lablab album semen], *Atractylodes macrocephala* Koidz. [Asteraceae; Atractylodis macrocephalae rhizoma], *Smilax glabra* Roxb. [Smilacaceae; smilacis glabrae rhizoma], *Glycyrrhiza uralensis* Fisch. [Fabaceae; Glycyrrhizae radix], *Platycodon grandiflorum* (Jacq.) A.DC. [Campanulaceae; Platycodonis radix], *Nelumbo nucifera* Gaertn. [Nelumbonaceae; Nelumbinis semen], *Panax ginseng* C.A.Mey. [Araliaceae; Ginseng radix et rhizoma], *Amomum villosum* Lour	NAFLD	Male SD rats/Male Wistar rats	Enhance liver perfusion; Improve hepatocyte morphology	Tang et al. (2020), Pan et al. (2021)
	[Zingiberaceae; Amomi fructus], *Dioscorea opposita* Thunb. [Dioscoreaceae; dioscoreae rhizoma], *Coix lacryma-jobi L.* var. *mayuen* (Roman.) Stapf [Poaceae; Coix seeds]				
Si-Ni-San	*Bupleurum chinense* DC. [Apiaceae; Bupleuri radix], Paeonia lactiflora Pall. [Paeoniaceae; paeoniae rubra radix]. *Citrus aurantium* L. [Rutaceae; aurantii pericarpium], *Glycyrrhiza uralensis* Fisch. [Fabaceae; Glycyrrhizae radix]	Hepatic fibrosis	Male C57BL/6J mice	Inhibit angiogenesis; Inhibit activation of HSCs and ECM accumulation	Wang et al. (2021)
Xia-Yu-Xue decoction	*Rheum officinale* Baill. [Polygonaceae; Rhei radix et rhizoma], *Prunus persica* (L.) Batsch. [Rosaceae; persicae semen], *Eupolyphaga sinensis* Walker [Eupolyphaga; Female whole insect]	Hepatic fibrosis	Male Wistar rats/Male C57BL/6J mice	Inhibits the angiogenesis and the activation of HSCs	Du et al. (2011)
Xue-Fu-Zhu-Yu decoction	*Prunus persica* (L.) Batsch. [Rosaceae; persicae semen], *Carthamus tinctorius* L. [Asteraceae; Carthami flos], *Angelica sinensis* (Oliv.) Diels. [Apiaceae; Angelicae sinensis radix], *Rehmannia glutinosa* Libosch. [Orobanchaceae; rehmanniae radix], *Achyranthes bidentata* Bl. [Amaranthaceae; Achyranthis radix], *Ligusticum chuanxiong* Hort. [Apiaceae; Chuanxiong rhizoma], *Platycodon grandiflorus* (Jacq.) A.DC. [Campanulaceae; Platycodonis radix], *Paeonia lactiflora* Pall. [Paeoniaceae; Paeoniae rubra radix], *Citrus aurantium* L. [Rutaceae; Aurantii pericarpium], *Glycyrrhiza uralensis* Fisch. [Fabaceae; Glycyrrhizae radix], *Bupleurum chinense* DC. [Apiaceae; Bupleuri radix]	Hepatic fibrosis	Male C57BL/6J mice	Antiangiogenic effect; Protect the functionality of LSECs	Zhou et al. (2014)
Tao Ren-Hong Hua herb pair	*Prunus persica* (L.) Batsch. [Rosaceae; Persicae semen], *Carthamus tinctorius* L. [Asteraceae; Carthami flos]	Chronic liver disease	Equal numbers of male and female KM mice	Inhibit pathological liver angiogenesis; Anti-inflammatory	Xi et al. (2016)
Da-Huang-Zhe-Chong pill	*Rheum officinale* Baill. [Polygonaceae; Rhei radix et rhizoma], *Rehmannia glutinosa* Libosch. [Orobanchaceae; rehmanniae radix], *Cutellaria baicalensis* Georgi. [Lamiaceae; Scutellariae baicalensis radix], *Paeonia lactiflora* Pall. [Paeoniaceae; Paeoniae radix], *Glycyrrhiza uralensis* Fisch. [Fabaceae; Glycyrrhizae radix], *Prunus armeniaca L. var. ansu* Maxim [Rosaceae; Armeniacae semen], *Prunus persica* (L.) Batsch. [Rosaceae; persicae semen], *Toxicodendron vernicifluum (Stokes) F.A.*Barkl. [Anacardiaceae; Toxicodendri resina], *Eupolyphaga sinensis* Walker [Eupolyphaga; Female whole insect], *Whitmania pigra* Whitman [Piscicolidae; Whole worm], *Tabanus bivittatus* Matsumura [Tabanidae; Female insect body], *Holotrichia diomphalia* Bates [Scarabaeoidea; Whole Insect]	HCC	Male SD rats	Improve hepatic sinusoidal capillarization; Regulate the balance of sinusoidal dilation and contraction; Reduce portal vein pressure and collagen fiber deposition	Fu et al. (2023)
Yi-Guan-Jian decoction	*Glehnia littoralis* Fr. Schmidt ex Miq. [Apiaceae; glehniae radix], *Ophiopogon japonicus* (L. f.) Ker-Gawl. [Asparagaceae; ophiopogonis radix], *Angelica sinensis* (Oliv.) Diels. [Apiaceae; Angelicae sinensis radix], *Rehmannia glutinosa* Libosch. [Orobanchaceae; rehmanniae radix], *Lycium barbarum* L. [Solanaceae; lycii fructus], *Melia toosendan Sieb.et Zucc*. [Meliaceae; meliae fructus]	Hepatic cirrhosis	Male C57BL/6J mice	Anti-angiogenic effect	Zhou et al. (2015)
Jie-Du-Hua-Yu granule	*Paeonia lactiflora* Pall. [Paeoniaceae; paeoniae rubra radix], *Artemisia scoparia Waldst.et Kit*. [Asteraceae; artemisiae herba], *Rheum officinale* Baill. [Polygonaceae; Rhei radix et rhizoma], *Curcuma phaeocaulis Val*. [Zingiberaceae; curcumae radix], *Scleromitrion diffusum* (Willd.) R. J. Wang [Rubiaceae; hedyotidis diffusae herba], *Acorus tatarinowii* Schott [Acoraceae; acori gramineri rhizoma]	ALF	Male Wistar rats	Negative regulation of angiogenesis; Fibrinolysis; Regulation of cell shape	Qiu et al. (2019)
Yu-Ping-Feng-San	*Astragalus membranaceus* (Fisch.) Bge. var. *mongholicus* (Bge.) Hsiao [Fabaceae; astragali radix], *Atractylodes macrocephala* Koidz. [Asteraceae; Atractylodis macrocephalae rhizoma], *Saposhnikovia divaricata* (Turcz.) Schischk. [Apiaceae; saposhnikoviae radix]	HCC	Male C57BL/6J mice	Anti-angiogenesis effect	Yuan et al. (2019)
Jie-du recipe	*Cremastra appendiculata* (D.Don) Makino [Orchidaceae; cremastrae tuber], *Actinidia valvata* Dunn [Actinidiaceae; Actinidia radix], *Salvia chinensis* Benth. [Lamiaceae; Salvia herba], *Gallus gallus domesticus* Brisson [Phasianidae; Chicken gizzard membrane]	HCC	Human HCC cell line Huh 7; Human immortalized endothelial cells EA.hy 926	Inhibit hypoxia-induced angiogenesis	Lin et al. (2021)

**TABLE 3:** Active metabolites with potential activity in regulating hepatic microcirculatory disturbance.

Metabolite names	TCM formula	Disease	Mouse/patient/cell	Therapeutic effects	Reference
Caffeic acid (1)	*Salvia miltiorrhiza* Bge. [Lamiaceae; Salviae miltiorrhizae radix et rhizoma]	HIRI	Male SD rats	Increase blood flow velocity and perfusion volume; Improve liver lobule structure; Anti-inflammatory	Mu et al. (2015), Mu et al. (2018)
Plumbagin (2)	*Plumbago zeylanica* L. [Plumbaginaceae; *Plumbago* radix et folium]	ALF	Female ICR mice	Reduce thrombus formation; Anti-inflammatory	Wang et al. (2016)
Berberine (3)	*Coptis chinensis* Franch. [Ranunculaceae; Coptidis rhizoma]	HIRI	Male SD rats	Reduce liver lobular edema and hemorrhage; Improve liver lobule structure; Anti-inflammatory	Sheng et al. (2015)
Acteoside (4)	*Lantana camara* L. [Verbenaceae; Lantana radix, folium and flos]	HIRI	Mice	Ameliorate characteristic sinusoidal congestion, vacuolization, hepatocytes necrosis, and evident oxidative stress; Reversed the senescent fate of LSECs	Jia et al. (2023)
Amarogentin (5)	*Swertia davidii* Franch. [Gentianaceae; Swertia davidii Franch herba]	HCC	HepG2 and Huh7 cell lines, male BALB/c nu/nu mice	Inhibit angiogenesis	Zhang et al. (2020)
Levistilide A (6)	*Angelica sinensis* (Oliv.) Diels. [Apiaceae; Angelicae sinensis radix]	Hepatic fibrosis	Male Wistar rats	Antiangiogenesis; Alleviating sinusoid capillarization	Zhao et al. (2017)
Hydroxysafflor yellow A (7)	*Carthamus tinctorius* L. [Asteraceae; Carthami flos]	HCC	H22 tumor-bearing mice	Inhibit angiogenesis	Yang et al. (2015), Zhang et al. (2019a)
Oroxylin A (8)	*Cutellaria baicalensis* Georgi. [Lamiaceae; Scutellariae baicalensis radix]	Hepatic fibrosis	Male ICR mice	Prevent angiogenesis of LSECs	Zhang et al. (2018)
Curcumol (9)	*Curcuma longa* L. [Zingiberaceae; Curcumae longae rhizoma]	Hepatic fibrosis	Male SD rats/Male ICR mice	Returne the microcirculation in liver; Improve Sinusoidal capillarization	Zheng et al. (2020)

## 6 Summary and outlook

This review provides updated insights into the pathogenic mechanisms underlying hepatic microcirculatory disturbance and the associated detection techniques. It also summarizes the characteristics of hepatic microcirculatory disturbance in various liver diseases and elucidates the regulatory effects of TCM. Therefore, hepatic microcirculatory disturbance plays a crucial role in the pathogenesis of liver diseases and may become an effective approach for the future treatment of liver diseases.

Although significant strides have been made in understanding the mechanisms underlying hepatic microcirculatory disturbance, numerous issues still need to be resolved. Firstly, the detection techniques for hepatic microcirculation cover histopathology, microcirculation detection, and advanced genomic technologies. While these techniques play an important role in detecting hepatic microcirculation, the diversity of detection methods may lead to a lack of standardization, complicating research results. Additionally, it remains unclear whether there are specific pathological changes in hepatic microcirculatory disturbance caused by different etiologies, which requires further research to uncover characteristic markers. Secondly, although various treatment methods are available, their efficacy and safety remain uncertain, especially in long-term management and individualized treatment. More clinical trials and research data are needed to guide clinical practice. Lastly, early diagnosis and prevention of hepatic microcirculatory disturbance are pressing issues, but effective early screening tools and strategies are currently lacking. Therefore, developing new early diagnostic techniques and preventive measures will have a profound impact on improving the quality of life for patients with liver diseases.

Hepatic microcirculatory disturbance is an essential factor leading to the occurrence, development, and worsening of liver disease. It is also the key to preventing and treating liver disease with TCM. TCM emphasizes holistic treatment and syndrome differentiation. It is often used as an alternative or complementary therapy, and combined with Western medicine to maximize the therapeutic effects. TCM shows advantages in treating hepatic microcirculatory disturbance due to its multi-metabolites, multi-targets methods that regulate hepatic hemodynamics and maintain microcirculatory homeostasis. Nevertheless, the application of TCM in treating hepatic microcirculatory disturbance faces several challenges and limitations. Firstly, the complexity of its metabolites poses difficulties in isolating and verifying active metabolites. Secondly, the small sample sizes of TCM clinical trials and lake strict control groups of have led to a lack of clinical evidence. Furthermore, the placebo effect and patient expectancy can also potentially skew the outcomes of some studies. Given the variability in TCM formulas and dosages, efforts to standardized treatment protocols for hepatic microcirculatory disturbance are crucial. To address cultural and regulatory challenges in TCM clinical trials for hepatic microcirculatory disturbance, it’s essential to enhance international cooperation and communication to overcome cultural differences. Additionally, working closely with regulatory agencies and adhering to laws and regulations will improve clinical trials compliance.

In summary, although significant progress has been made in the study of hepatic microcirculatory disturbance, many unknown areas still require further exploration. Future research should employ multidisciplinary collaboration and innovative technologies such as genomics, proteomics, and metabolomics to comprehensively elucidate the molecular mechanisms of hepatic microcirculatory disturbance. Additionally, the use of emerging research tools, such as organoid models (Panwar et al., 2021) to simulate the microenvironment of the human liver will facilitate the pathophysiological research of hepatic microcirculatory disturbance as well as the study of the efficacy and mechanisms of TCM formula and active metabolites, potentially addressing the limitations of traditional clinical research. In addition, utilizing liver-targeted drug delivery systems, such as passive and active targeted drug delivery systems, as well as the physicochemical strategies for targeted drug delivery, can ensure the precise delivery of TCM to the liver (Ma et al., 2019). Finally, exploring new therapeutic strategies, such as gene therapy and cell therapy, promote the development of TCM in treating hepatic microcirculatory disturbance, and enhance the prognosis and quality of life for patients.

## Figures and Tables

**Table:** 

**Ach**	Acetylcholine
**ALD**	Alcoholic liver disease
**ALF**	Acute liver failure
**Ang II**	Angiotensin II
**CCL2**	Chemokine (C-C motif) ligand 2
**CCl4**	Carbon tetrachloride
**ECM**	Extracellular matrix
**eNOS-NO-cGMP**	Endothelial nitric oxide synthase-nitric oxide-cyclic guanosine monophosphate
**ERK**	Extracellular regulated protein kinases
**ET-1**	Endothelin-1
**FGF**	Fibroblast growth factor
**GM-CSF**	Granulocyte-macrophage colony-stimulating factor
**HBV**	Hepatitis B virus
**HCC**	Hepatocellular carcinoma
**HCV**	Hepatitis C virus
**H&E**	Hematoxylin and eosin
**HIF-1α**	Hypoxia-inducible factor-1α
**HIRI**	Hepatic ischemia-reperfusion injury
**HSCs**	Hepatic stellate cells
**ILs**	Interleukins
**I/R**	Ischemia-reperfusion
**KCs**	Kupffer cells
**LECT2**	Leukocyte cell-derived Chemotaxin 2
**LPS**	Lipopolysaccharide
**LSECs**	Liver sinusoidal endothelial cells
**MAPK**	Mitogen-activated protein kinase
**MCP-1**	Monocyte chemoattractant protein-1
**MK**	Midkine
**MMP**	Matrix metalloproteinase
**NAFLD**	Non-alcoholic fatty liver disease
**NF-κB**	Nuclear factor-κB
**NGF**	Nerve growth factor
**NO**	Nitric oxide
**PDGF**	Platelet-derived growth factor
**PI3K**	Phosphatidylinositol-3-kinase
**PG**	Prostaglandin
**RER**	Rough endoplasmic reticulum
**ROS**	Reactive oxygen species
**SEM**	Scanning electron microscope
**Smad3**	Mothers against decapentaplegic homolog 3
**SMI**	Super microvascular imaging
**TCM**	Traditional Chinese medicine
**TEM**	Transmission electron microscopy
**TGF**	Transforming growth factor
**TLR**	Toll-like receptor
**TNF-α**	Tumor necrosis factor-α
**TrkA**	Tropomyosin receptor kinase A
**TSLP-STAT3**	Thymic stromal lymphopoietin-signal transducer and activator of transcription 3
**TSP-1**	Thrombospondin-1
**TXA2**	Thromboxane A2
**VEGF**	Vascular endothelial growth factor
